# Potentially Inappropriate Medications and Drug–Drug Interactions in Geriatrics Cardiac Patients Visiting to Tertiary Care Central Hospital of Nepal

**DOI:** 10.1002/agm2.70034

**Published:** 2025-08-14

**Authors:** Roshan Giri, Rajeev Shrestha, Aatma Rai, Gobinda Kandel, Punam Gauchan, Parbati Thapa

**Affiliations:** ^1^ Pharmaceutical Sciences Program, School of Health and Allied Sciences Pokhara University Pokhara Nepal; ^2^ Department of Pharmacy Bharatpur Hospital Bharatpur Nepal; ^3^ NIHR Newcastle Patient Safety Research Collaboration Newcastle University Newcastle UK; ^4^ Department of Medicine Bharatpur Hospital Bharatpur Nepal

**Keywords:** beers criteria, cardiac patients, drug–drug interaction, geriatrics, Nepal, potentially inappropriate medication

## Abstract

**Objective:**

Potentially Inappropriate Medications (PIMs) and Drug–Drug Interactions (DDIs) among geriatrics are a prominent issue and place a considerable burden on the quality health outcome. This study aimed to assess PIMs and DDIs particularly among geriatric cardiac elderly patients attending the outpatient department of a tertiary care hospital of Nepal.

**Methodology:**

A prospective cross‐sectional study was conducted on geriatrics patients (≥ 65 years) attending the cardiology out‐patient department of Bharatpur Central Hospital, Nepal. Beers criteria were used to assess PIM, and Medscape software was employed to assess DDIs. Regression analysis was performed to identify the predictors of PIM and DDIs experienced by patients.

**Results:**

A total of 140 geriatric patients were enrolled in the study. A total of 683 drugs were prescribed to them, with an average of 4.59 medications per patient. 23.6% and 65% of patients were found to be prescribed PIMs and encountered DDIs, respectively. Proton Pump Inhibitors (PPI) (66.67%) were the most prescribed PIMs and involved in identified DDIs. The removal of PIMs was calculated to reduce 11.72% of the cost of patients prescribed PIMs and 19.88% of identified DDIs. The number of medications prescribed was found to be a significant predictor for PIM (Odd Ratio: 1.352) and DDIs (Odd Ratio: 2.217) encountered.

**Conclusion:**

This study highlights the concerning prevalence of potentially inappropriate medication and drug–drug interactions among ambulatory cardiac elderly patients. Integration of regular medication audits and evidence‐based prescribing practices is essential to optimize pharmacological management and enhance patient safety.

## Graphical abstract

Elderly cardiac patients are usually taking multiple medications. These cause them to encounter potentially inappropriate medications and drug–drug interactions, which increase the risk of adverse health outcomes as well as the economic burden caused by them. Identifying the prevalence and significant predictors of encountering DDIs and PIMs in patients with potential benefits of discontinuing inappropriate medications contributes to adopting a proper system to improve care for elderly patients. In this study, we investigate the prevalence of PIMs and DDIs encountered by elderly cardiac patients visiting the cardiology outpatient department of Bharatpur Central Hospital, Nepal. The Beers criteria were used to identify PIMs, and Medscape software was employed to evaluate DDIs. We identify the considerable prevalence of PIMs and DDIs with prevalent inappropriate medication (Proton pump inhibitors). Furthermore, the deprescribing of inappropriate medication appeared to reduce one‐fifth of total drug interactions and 10% of total medication costs. Similarly, the number of medications prescribed showed to increase the risk of PIMs and DDIs significantly. Moreover, low literate patients were found to be significantly associated with PIMs risk, demanding closed care to them. This study underscores the integration of regular medication audits and evidence‐based prescribing practices to enhance medication safety and optimize healthcare outcomes in geriatric populations in Nepal.

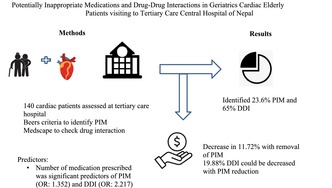

## 1 Introduction

Globally, the aging population is increasing. Particularly in low‐ and middle‐income countries, it is estimated to have more than two‐thirds of the global older aged population by 2050 [1]. In Nepal, the 2021 census showed 10.21% of the ≥ 60 aged population with a growth rate of 3.29% [2]. With the age‐related changes in physiology and pharmacokinetics, pharmacotherapy in this population can be complex, demanding tailored drug therapy [3].

Older people are prone to be prescribed multiple medications due to multi‐morbid conditions, resulting in a higher probability of receiving potentially inappropriate medication (PIM) and drug–drug interactions (DDIs) [4]. PIMs are those medications that produce more harmful effects than beneficial ones to patients [4]. A recent 2023 systematic review showed that globally, 37.4% of older people receive PIM, with the highest prevalence among Africans (47.0%), followed by South Americans (46.9%) and Asians (37.2%) [5]. Similarly, 28.8% of older people reported experiencing DDI globally [6]. Few studies reported in Nepal also found PIMs, ranging from 5% to 24% [7, 8, 9, 10, 11], and DDIs, ranging from 1.4 to 3.3 per patient [12, 13], experienced by the elderly population. Moreover, the occurrence of PIM and DDIs was found to be associated with a substantial number of adverse drug events (ADR), increased hospital stays, decreased quality of life, and increased healthcare costs [14, 15]. Additionally, the prescription of inappropriate medications has been linked to increased morbidity and mortality [16].

Several studies conducted so far have found the prescribing of PIMs and experiencing DDIs in patients with cardiovascular diseases with a range of 7% to 85% [17, 18]. Cardiovascular diseases are one of the global health problems causing almost one third of all the deaths globally [19]. Similarly, it was reported as causing 26.9% of total deaths and 12.9% of total disability in Nepal [20]. Despite a few studies documenting PIMs and DDIs patterns in Nepal [7, 8, 9, 10, 11, 12, 13], there are no studies documenting the inappropriate medication utilization and drug–drug interaction prevalence among the cardiac elderly population in Nepal.

Therefore, we aimed to assess the pattern of PIM and drug–drug interaction among patients visiting the cardiac out‐patient department in Nepal. Furthermore, this study explored factors associated with them to provide a better understanding to develop appropriate care for elderly cardiac patients. Overall, this study aimed to illustrate the context of elderly patients' medication‐related problems from the context of a developing country to advocate globally in enhancing the quality of medicinal care to improve aging patients' health.

## 2 Methods

### 2.1 Study Design and Setting

It was a prospective cross‐sectional study conducted in the cardiac outpatient department of Bharatpur Hospital from Dec 2023 to June 2024, a tertiary care central hospital in central Nepal. The hospital is usually the first point of service for nearby people in 10 districts and has a specialized geriatrics unit. The Strengthening the Reporting of Observational Studies in Epidemiology (STROBE) Statement was compiled in reporting this study [21].

### 2.2 Ethics Statement

Ethical approval for this study was obtained from the Institutional Review committee of Pokhara University (Ref no. 83/2081/2082). Respective hospital approval was also obtained before conducting this study at Bharatpur Hospital (Ref no. 1646). Informed consent was taken prior to data collection. All participants were explained about the study.

### 2.3 Study Sample and Sampling

A prevalence‐based sample size calculation approach was taken for this study as there were two previous studies which reported the prevalence of PIM among elderly patients visiting the out‐patient department of a tertiary care hospital resembling the context of these current study settings [8, 11]. A total of 125 sample size was calculated considering the 95% confidence level and 7.5% margin of error, with an estimated prevalence of 24%. Considering the potential non‐response, 140 patients were estimated to be approached in this study. Patients visiting the hospital pharmacy for their medications were purposively selected and approached to participate.

Patient aged ≥ 65 years of both genders with an initial diagnosis visiting Cardiology OPD, medication refills, and those with a governmental insurance scheme were enrolled in the study. Those who had mental illness and refused to participate in the study were excluded from the study.

### 2.4 Study Tool and Data Collection

The study tool for this study was divided into three parts. First is socio‐demographic and medication‐related characteristics of the participants, such as their age, gender, and educational level, presence of comorbid conditions, primary diagnosis, and medication prescribed. Second is the information on the potentially inappropriate medication. The 2023 updated Beers criteria provided a list of medication considered potentially inappropriate to use in older adults, which was taken to identify potentially inappropriate medications [22]. The third part was about the cost of prescribed medication.

For the calculation of medication cost, the reference price of medication provided by the health insurance scheme of the government social security program was taken in case of medications dispensed from the insurance scheme to patients [23]. In case of dispensing medication on cash, the cost (maximum retail price) incurred by the patients was taken. Information on drug interaction of prescribed medication was collected checking Medscape, an online free drug–drug interaction checking portal [24].

### 2.5 Data Collection Procedure

Patient's data were collected at the special geriatric patient unit at the hospital pharmacy outlet of the hospitals. Patients visiting the hospital pharmacy were approached to participate in the study. Upon receiving their written and verbal consent, data were collected from them. Information on patient's sociodemographic characteristics were obtained by interviewing them, and their clinical diagnosis and medication‐related information were obtained by reviewing their prescription.

### 2.6 Data Management and Analysis

Data obtained from the data collection tool were coded and entered in SPSS version 29. The cost of each drug was recorded in Excel. Descriptive statistics were applied to present the sociodemographic profiles and pattern of PIM and DDIs in cardiac elderly patients. Binary logistic regression analysis was used to assess the factors associated with the patients encountering PIMs and DDIs. A *p* ≤ 0.005 was considered statistically significant.

## 3 Results

### 3.1 Characteristics of Participants and Their Prescribed Medications

Majority of the participating patients were female (53.9%), illiterate (60.7%), with a mean age of 73.29. Hypertension was the most common primary diagnosis (58.6%) followed by ischemic heart disease (17.1%), dyslipidaemia (12.9%) and coronary artery disease (4.3%). Most of the patients had comorbid conditions (59.3%, *n* = 83), where diabetes mellitus (*n* = 78) and pulmonary disease (*n* = 64) were very common.

A total of 683 drugs were prescribed to 140 patients with an average of 4.59 medicines. 41% (*n* = 57) of patients had polypharmacy (5 to 9 prescribed medicines) and 6% (*n* = 9) patients had hyper‐polypharmacy (≥ 10 prescribed medicines). The most prescribed medication was calcium channel blocker (43.57%), followed by HMG‐COA reductase inhibitors (40.71%), Beta Blockers (40%), and Angiotensin Receptor Blocker (ARB) (30.71%). Proton Pump Inhibitors (PPIs) (56.42%) were the most prescribed non‐cardiac medications, followed by Oral Hypoglycaemic Drugs (51.42%) and Vitamins Supplements (39.28%).

### 3.2 Potentially Inappropriate Medication Prescribed to Elderly Cardiac Patients

The review of the prescribed medicines using 2023 updated Beers criteria identified 33 prescriptions with at least one PIM, giving the prevalence rate of 23.6%. Proton Pump Inhibitors were the most prescribed PIM (*n* = 22, 66.67%), followed by Rivaroxaban (*n* = 3, 9.09%) and Digoxin (*n* = 2, 6.06%). A total of 9 types of PIM were found in this study. Table 1 provides the detail of PIM identified.

**TABLE 1:** Potentially inappropriate medication uses in older adults as per 2023 American Geriatrics Society Beers Criteria [22].

Medicine name (number, %)	Recommendation	Quality of evidence	Strength of recommendation
**Antihistamines (1, 3.03%)**			
Cyproheptadine (1, 3.03%)	Avoid	Moderate	Strong
**Cardiovascular and antithrombotic (5, 15.15%)**			
Rivaroxaban (3, 9.09%)	Avoid for long‐term treatment of atrial fibrillation or VTE in favor of safer anticoagulant alternatives.	Moderate	Strong
Digoxin (2, 6.06%.)	Avoid this rate control agent as first line therapy for atrial fibrillation. Avoid as first‐line therapy for heart failure. If used for atrial fibrillation or heart failure, avoid dosages > 0.125 mg/day	Atrial fibrillation; heart failure: low dosage > 0.125 mg/day: moderate	Strong
**Gastrointestinal (22, 66.67%)**			
Pantoprazole (16, 48.49%)	Avoid scheduled use for > 8 weeks unless for high‐risk patients (e.g., oral corticosteroids or chronic NSAID use), erosive esophagitis, Barrett esophagitis, pathological hyper secretory condition, or demonstrated need for maintenance treatment	*C. difficile* , bone loss, and fractures: high pneumonia and GI malignancies: moderate	Strong
Esomeprazole (2, 6.06%)			
Rabeprazole (4, 12.12%)			
**Endocrine (sulphonylureas) (4, 12.12%)**			
Gliclazide (2, 6.06%)	Avoid sulphonyl urea as first or second line monotherapy or add on therapy unless there are substantial barriers to the use of safer and effective agents	Hypoglycaemia: high CV events and all‐cause mortality: moderate; CV death and ischemic stroke: low	Strong
Glimepiride (2, 6.06%)			Strong
**Central nervous system (1, 3.03%)**			
Clonazepam (1, 3.03%)	Avoid, Risk of abuse misuse and addiction. May lead to clinically significant physical dependence.	Moderate	Strong

*Note*: Total (*n* = 33, 100%).

Table 2 provides the information on the distribution of potentially inappropriate medication across patients' sociodemographic characteristics and regression analysis to identify significantly associated factors. The number of medications prescribed was significantly associated with PIM occurrence. For each additional medication, the odds of being prescribed a PIM increased by 35.2%. Similarly, patients with a primary level of education were significantly more likely to be prescribed PIMs, with 3.359 times higher odds compared to illiterate patients.

**TABLE 2:** PIM across sociodemographic characteristics and regression analysis.

Socio demographic characters		PIM prescribed		*p*	Odd ratio	95% confidence interval	
		No (%)	Yes (%)			Lower	Upper
Age (minimum 65, maximum 98, mean 73.29, SD 7.071)				0.159	1.051	0.981	1.126
Gender	Male	53 (80.3)	13 (19.7)	Reference			
	Female	54 (73)	20 (27)	0.188	1.833	0.744	4.512
Educational Level	Illiterate	69 (81.2)	16 (18.8)	Reference			
	Primary	31 (64.6)	17 (35.4)	**0.015**	3.359	1.264	8.929
	Secondary	7 (100)	0 (0)	0.999	0.000	0.000	
Presence of comorbid conditions	No	45 (78.9)	12 (21.1)	Reference			
	Yes	62 (74.7)	21 (25.3)	0.412	0.667	0.253	1.755
Number of prescribed medicines (minimum 1, maximum 13, mean 4.59, SD 2.608)				**0.001**	1.352	1.129	1.619
Constant				0.018	0.001		

Table 3 described the cost of prescribed medications including PIM to cardiac elderly patients. The total prescription cost for 3 months was found to be Rs. 667,496 (Rs. 4767.82 per patient). Further, the cost of prescriptions for those patients with PIM was found to be Rs. 227,075 (Rs. 6881.06 per patient). Similarly, the overall cost of PIM was found to be Rs. 26,620. On further analysis, the proportion of PIM cost to total prescribed medication cost was 3.99 while undertaking all kinds of patients and 11.72 while undertaking PIM‐receiving patients only. Mann–Whitney *U* test showed a significant difference in medication cost between patients with PIM and without PIM.

**TABLE 3:** Description on cost of PIM prescribed to cardiac elderly patients (for 3 months).

SN	Characteristics	Patients without PIM	Patients with PIM	Total patients
1	Minimum cost	450	780	450
2	Maximum cost	23,372	18,060	23,372
3	Median cost (IQR)	2974 (4406)	5525 (8358)	3244 (5015)
4	Total cost	440,421	227,075	667,496

*Note*: Mann–Whitney *U* test. Test statistics: 1153, *p*‐value: 0.003.

### 3.3 Drug–Drug Interaction on Prescribed Medications to Cardiac Elderly Patients

Altogether 65% (*n* = 91) patients were found to have 337 all kinds of drug interactions (minor, monitor closely and serious), while monitor closely and serious types of drug interaction appeared in 77 patients. A maximum of 17, 15, and 14 unique drug interactions were found among three patients who were prescribed 9, 11, and 11 medicines, respectively. Although the most found drug interaction was a minor type between pantoprazole and levothyroxine (5.34%), monitor closely types of drug interaction were found in the majority of patients (*n* = 76) with a total 270 interactions. Table 4 provides the detail of the top five drug interactions under three types of DDIs.

**TABLE 4:** Top five DDIs under three categories of DDI as per Medscape.

S.N.	Drug–drug interaction	Interaction detail
**A. Serious (16, 4.75%) occurred in 13 patients**		
1	Aspirin + Ramipril (3, 0.89%)	Pharmacodynamic antagonism. Avoid or Use Alternate Drug. Coadministration may result in a significant decrease in renal function. NSAIDs may diminish the antihypertensive effect of ACE inhibitors. The mechanism of these interactions is likely related to the ability of NSAIDs to reduce the synthesis of vasodilating renal prostaglandins.
2	Bisoprolol + Digoxin^a^ (2, 0.59%)	Pharmacodynamic synergism. Bisoprolol increases the effects of digoxin. Increases the risk of Bradycardia.
3	Clopidogrel + Esomeprazole^a^ (2, 0.59%)	Esomeprazole reduces the effect of clopidegrol by affecting hepatic enzyme CYP2C19 metabolism which is responsible for formation of active metabolite once metabolized by CYP2C19.
4	Clopidogrel + Rabeprazole^a^ (2, 0.59%)	Rabeprazole reduces the effect of clopidegrol by affecting hepatic enzyme CYP2C19 metabolism which is responsible for formation of active metabolite once metabolized by CYP2C19.
5	Digoxin^a^ + Esomeprazole^a^ (2, 0.59%)	Esomeprazole increases the level or effect of digoxin by increasing gastric P^H^
**B. Monitor Closely (270, 80.12%) occurred in 76 patients**		
1	Amlodipine + Metformin (17, 5.04%)	Amlodipine decreases the effect of metformin by pharmacodynamics antagonism.
2	Furosemide + Spironolactone (16, 4.75%)	Spironolactone increases and furosemide decreases the serum potassium level.
3	Losartan + Metoprolol (12, 3.56%)	Pharmacodynamics synergism. Losartan and metoprolol both increases the serum potassium levels.
4	Aspirin + Losartan (10, 2.97%)	Aspirin decreases the effect of losartan by pharmacodynamics antagonism. They either increase the toxicity of other drug which results in renal function deterioration.
5	Atenolol + Amlodipine (10, 2.97%)	Pharmacodynamic Synergism. They both decrease blood pressure.
***C. minor* (51, 15.13%) in 37 patients**		
1	Levothyroxine + Pantoprazole^a^ (18, 5.34%)	Pantoprazole decreases the effects of levothyroxine by increasing the gastric P^H^.
2	Cyanocobalamin + Pantoprazole^a^ (6, 1.78%)	Pantoprazole decreases level of cyanocobalamin by inhibiting GI absorption.
3	Furosemide + Levothyroxine (6, 1.78%)	Furosemide increases the toxicity of levothyroxine. High dose of furosemide may inhibit binding of thyroid hormones to carrier proteins and result in transient increase in free thyroid hormones, followed by overall decrease in total thyroid hormones.
4	Cyanocobalamin + Metformin (4, 1.19%)	Metformin decreases the level of cyanocobalamin by unspecified interaction mechanism.
5	Hydrochlorothiazide + Metformin (4, 1.19%)	Pharmacodynamic antagonism. Hydrochlorothiazide will increase the level or effect of metformin by basic cationic drug competition for renal tubular clearance.
	Total	337 (100.00%)

^a^ Potentially inappropriate medication in older adults.

Table 5 provides the information on the distribution of drug–drug interactions (monitor closely and serious) across patients' sociodemographic characteristics and regression analysis to identify significantly associated factors. The number of medications prescribed was significantly associated with the occurrence of monitor closely and serious types of DDIs. For each additional medication, the odds of occurring DDIs increased by approximately 2.2 times.

**TABLE 5:** Drug–drug interaction (serious and monitor) across sociodemographic characteristics and regression analysis.

Socio demographic characters		Presence of serious or monitor closely DDI		*p*	OR	95% confidence interval	
		No (%)	Yes (%)			Lower	Upper
Age (minimum 65, maximum 98, mean 73.29, SD 7.071)				0.816	1.008	0.940	1.082
Gender	Male	26 (39.4)	40 (60.6)	Reference			
	Female	37 (50)	37 (50)	0.150	0.510	0.204	1.276
Educational level	Illiterate	38 (44.7)	47 (55.3)	Reference			
	Primary	21 (43.8)	27 (56.3)	0.924	0.954	0.360	2.529
	Secondary	4 (57.1)	3 (42.9)	0.892	0.866	0.110	6.829
Presence of comorbid conditions	No	29 (50.9)	28 (49.1)	Reference			
	Yes	34 (41)	49 (59)	0.038	0.332	0.117	0.942
Number of prescribed medicines (minimum 1, maximum 13, mean 4.59, SD 2.608)				**< 0.001**	2.217	1.637	3.003
Constant				0.665	0.278		

## 4 Discussion

Our study identified that 41% of elderly cardiac patients were experiencing polypharmacy with 4.59 average medications per patient. Most of the patients in our study were suffering from other comorbidities (59.3%) possibly supporting receiving multiple medications. Polypharmacy has been reported widely even in the previous studies conducted among ambulatory elderly patients in Nepal. A recent 2021 study showed polypharmacy in 34.5% of elderly patients who visited the hospital pharmacy [25] and 64.03% of elderly patients who visited community pharmacies [26]. Similar studies conducted in other developing countries such as Pakistan reported 72.3%, and Ethiopia reported 31.4% polypharmacy among cardiac elderly outpatients. The difference in prevalence might be the result of different healthcare settings, healthcare problems, and reporting time. However, the growing use of multiple medications among vulnerable elderly patients with multiple comorbidities necessitates the judicious use of medications and patient‐centered pharmaceutical care. Contrarily, the hospital pharmacist in Nepal is not well‐trained, and systems are not well‐supported to offer medication‐related closed services to patients [27].

Potentially Inappropriate Medications were found to be prescribed among 23.6% patients, which is similar to previous studies conducted among out‐patient geriatric patients (21.6%–26.3%) [8, 11]. This Nepalese prevalence is similar to the global prevalence of PIM among general elderly population (27.8%) [28]. However, PIM prevalence is lower compared to developing countries like Pakistan (67.4%) [29] and neighboring country Indian (29.3%) [30], which reported PIM in similar setting and group of patients, cardiac elderly outpatients. The portion of PIM could be higher in our study if other PIM identification criteria such as “in patients with certain disease condition”, “medication to be used with caution”, “potential inappropriate drug‐drug interaction” and “dosage adjusted in renal function” described in 2023 Beers Criteria [22], which was not accompanied in this study due the limitation of accessing to the patient's data. If these criteria were explicitly undertaken the prevalence of inappropriate medication could be higher.

Additionally, regression analysis in this study showed 1.352 and 3.359 odds of increased PIM prescribing with an increasing number of prescribed medications and in patients with a primary level of education. Both statistically significant associated factors necessitated the need for careful action toward reducing the number of prescribed medications and providing thorough care to patients with lower literacy. Patients in Nepal are relatively less literate and highly dependent upon medical doctors primarily and then on pharmacy professionals about their medicinal and medical decisions [31, 32, 33]. All these highlight the need for enhanced professional medicinal care delivery; however, the limited manpower, increased workload, and deficient healthcare system arrangement are the key challenges to ensuring proper medicinal care in Nepal [27, 34, 35]. Secondly, the multiple medications use risk necessitates close monitoring and careful prescribing. A previous study among elderly patients in this same institution, Bharatpur Hospital, reported that majority of patients were willing to reduce their prescribed medications [36]. Therefore, this study highlights the importance of considering reducing or discontinuing inappropriate medications wherever possible to reduce the medication‐related burden on elderly patients.

This study further explored the PIMs prescribed where proton pump inhibitors were the most predominantly (66.67%) prescribed. Many studies of other countries have also reported prescribing PPIs as inappropriate medications [37, 38, 39]. As per the systematic reviews, 82% of PPIs were not prescribed following the evidence‐based guideline and it is safe deprescribing is possible [40, 41]. This highlights the significance of possible reduction of inappropriate medication with bringing significant impact on patient's health and medication cost reduction.

In this study, we also explored the possible cost coverage by potentially inappropriate medicine. The overall cost of PIM was found to be Rs. 26,620 for 3 months of medication, which is 3.99% of all patients' total medication cost (667,496) and 11.72% of PIM prescribed patients' total medication cost (227,075). This reflects the possibility of saving annually Rs. 106,480 and Rs. 760.57 per patient. The projection of medication cost saving through the deprescribing of inappropriate medication could be higher while incorporating the nationwide data on PIMs prescribing. This would provide significant financial saving to the government and patients, which can be utilized toward the development of quality healthcare services to reduce the medication‐related problems.

Our study identified the prevalence of DDI among 65% of elderly cardiac outpatients. There was no previous DDI assessment among similar patients in similar settings, but the study among hospitalized elderly patients showed variable prevalence (21.3%–69.3%) [12, 42]. Despite the difference in prevalence due to different study settings and the assessment tool applied, it shows the considerable drug interactions among the elderly population in Nepal. Moreover, a 2019 study among the same institution reported DDI among 19.1% of all types of outpatients [43], which further confirms the need for closer care for elderly patients. Specifically, 13 and 80 patients experienced serious and monitor closely types of drug interactions. As per the Medscape recommendation, the serious types of drug interaction must be avoided using alternative medications, and monitor closely types of interaction require close monitoring of therapeutic effects of medicine with the possibility of intervening to mitigate the interaction effects. This requires closer care and monitoring of patients' medication with proper medication counseling to manage the indicated interactions. However, currently, hospital pharmacists are very lowly involved in medication review and patient‐focused care but mainly in medication dispensing [27, 44]. Hospital pharmacy guidelines advocate for the enrollment of adequate pharmacy manpower based on the bed capacity of the hospital including clinical pharmacists [45]; however, the advocacy for the pharmacy services by recruited pharmacy professionals is not sufficiently discussed and hence not implemented in practice. Furthermore, the lack of continued professional development practices failed to equip pharmacy manpower for better pharmacy service in Nepal [27, 33].

On the other hand, the regression analysis showed that the occurrence of DDIs can be significantly reduced with the reduction of the number of prescribed medications. It means the removal of inappropriate medication not only improves healthcare outcomes and saves medication costs, but it showed simultaneously reducing the risk of drug interaction and its associated burden. Out of 9 PIM identified in this study, 6 PIMs were found to be involved in 81 drug interaction events, which was 19.88% of the total drug interactions identified in this study. Specifically, proton pump inhibitors were the most common medication involved in drug–drug interactions, which is potentially inappropriate medication. The use of PPI was found to be one of the contributing factors for interaction in older adults with cardiovascular diseases [18]. Moreover, its continued use particularly reported increasing the risk of cardiovascular disease [46, 47]. Hence, the drug optimization, including deprescribing wherever possible, is essential for reducing drug interaction‐causing medications, specifically inappropriate medications [48].

Particularly, the patients visiting the out‐patients department in Nepalese hospitals encountered multiple medical prescribers. Due to the older age with lower literacy and higher workload with limited manpower in government healthcare settings, effective communication between patients and prescribers is rare, which results in multiple medication prescribing. Furthermore, the lack of a properly equipped system and training programs for pharmacy professionals prevents the possibility of identifying the medication‐related problems faced by elderly chronic disease patients who are at high risk of polypharmacy, PIMs, and DDIs. Nevertheless, this study reported the pattern of inappropriate medication and drug–drug interactions among cardiac elderly out‐patients visiting a tertiary care hospital, presenting the possible implications and the interventions that should be taken to improve patients' health outcomes.

## 5 Strength and Limitations

This is the first study illustrating the inappropriate medication prescribed utilizing updated 2023 Beers criteria and drug–drug interaction patterns among Nepalese cardiac elderly patients to our best knowledge. The PIM prevalence in this study reflects only those medicines that were recommended as inappropriate in the elderly population. Medicines that were considered inappropriate based on other criteria were not covered in this study due to limitations in access to patient's data; hence, careful interpretation of data is essential. This is a single‐centre and cross‐sectional study, which limits the generalizability to the entire elderly cardiac population of the country. However, this study represented the scenario of medication‐related issues, particularly PIM and DDIs among Nepalese cardiac elderly populations.

## 6 Conclusion

This study highlights the concerning prevalence of potentially inappropriate medication and drug–drug interactions among ambulatory cardiac elderly patients. Proton pump inhibitors emerged as the most prescribed PIM, significantly contributing to DDIs. The number of medications prescribed showed to increase the risk of PIMs and DDIs significantly. Moreover, low literate patients were found significantly associated with PIMs risk, demanding close care for them. Discontinuation of inappropriate medication was found to effectively reduce medication cost burden and the prevalence of DDIs, signifying the importance of deprescribing intervention. This study underscores the integration of regular medication audits and evidence‐based prescribing practices with developing a proper system and equipping healthcare professionals to optimize pharmacological management and enhance patient safety.

## Author Contributions

R.G. and R.S. conceptualized and designed the study. R.G. and A.R. performed data collection. R.G. and R.S. conducted data analysis and data interpretation. R.G. and R.S. wrote the draft of the manuscript. P.G. and P.T. supervised and contributed to revising and finalizing the manuscript. G.K. contributed as a field supervisor and contributed to finalizing the manuscript. All authors read and approved the final version of the manuscript before submission.

## Conflicts of Interest

The authors declare no conflicts of interest.

## Data Availability

All the relevant data are included in this article. Further detailed data, if required, can be available from the corresponding author on reasonable requisition.
